# Fatal Outcome of Ruptured Pulmonary Hydatid Cyst

**Published:** 2018-02

**Authors:** Kambiz Sheikhy, Azizollah Abbasi Dezfuli, Saviz Pejhan, Farahnaz Sadegh Beigee

**Affiliations:** 1 Lung Transplantation Research Center(LTRC), National Research Institute of Tuberculosis and Lung Diseases (NRITLD), Shahid Beheshti University of Medical Sciences, Tehran, Iran; 2 Tracheal Diseases Research Center, NRITLD, Shahid Beheshti University of Medical Sciences. Tehran, Iran.

**Keywords:** Pulmonary hydatid cyst, Albendazole, Rupture

## Abstract

Most authors believe that the optimal treatment for pulmonary hydatid cyst is surgery. Albendazole has been used as a prophylactic measure for reducing recurrence rate but there are some controversies about this strategy. Some researchers have described the increased risk of spontaneous rupture of cysts following albendazole treatment. In this case report, we present a case of spontaneous rupture of pulmonary hydatid cyst with fatal outcome that may be the adverse cause of albendazole.

## INTRODUCTION

Hydatid cyst is an endemic disease in Iran and other Middle East countries. The lung is the second most common organ after liver that is involved in this parasitic disease. Although there are some controversies about the optimal therapeutic strategy for patients with pulmonary hydatid cyst, most of the investigators believe that surgery is the best therapy. Albendazole has been used as a prophylactic drug before surgery for reducing recurrence or as a definitive medical therapy in patients with this disease. Some physician uses this drug as a sole treatment for pulmonary hydatid cyst especially in patients who are not a good candidate for surgery (1–3). On the other hand, clinical observations have shown an increased incidence of spontaneous rupture of lung cysts following administration of albendazole. In this case report, we present a patient with multiple hydatid cysts in liver and lungs that had been treated with albendazole but his clinical course became grave due to rupturing of the pulmonary cysts and terminated with the death of the patient.

## CASE SUMMARY

**Figure 1.**
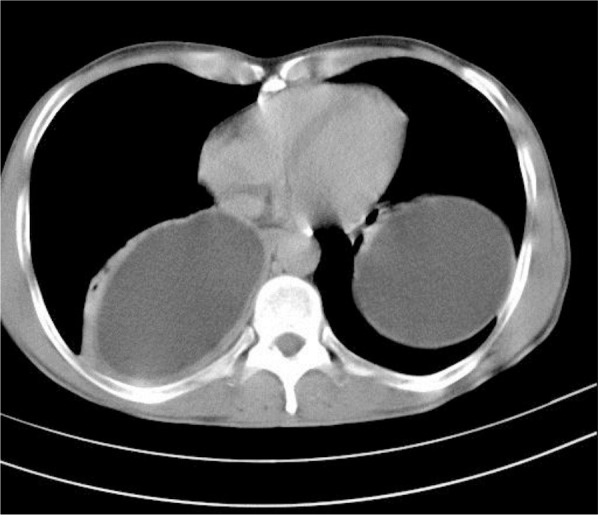
Chest CT scan shows bilateral and intact cysts.

A 43-years-old man was admitted to the emergency room due to severe cough, fever, vomiting and copious sputum production. Physical examination at arrival revealed tachypnea and bilaterally diminished breath sounds. Nine months before this event, the patient was visited by a physician due to right flank pain and based on the detection of a large cystic lesion on liver sonography, the diagnosis of hydatid cyst was made. At that time bilateral pulmonary hydatid cysts were discovered on chest X-ray that was approved by CT scan (Figure 1).

**Figure 2.**
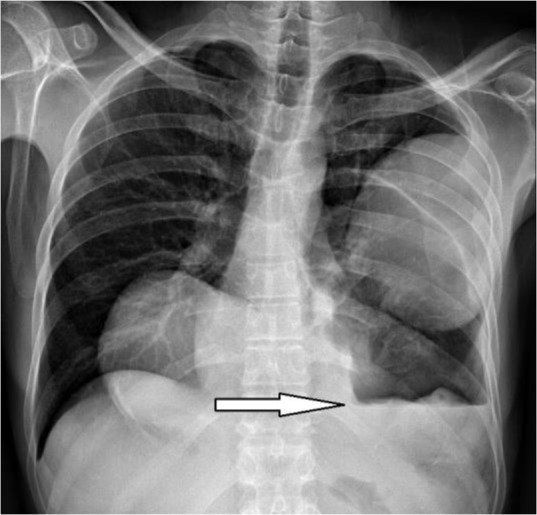
Chest X-ray shows left lung ruptured cyst (arrow).

Thoracotomy and resection of the pulmonary cyst were planned for treating the lung cysts and preoperative albendazole was begun with the dose of 400 mg twice daily. But patient postponed the surgery by himself and used albendazole for about 70 days. Two weeks after discontinuing albendazole there was an attack of a cough, fever and salty tasting sputum; however after 24 hours and following accentuation of symptoms he was referred to the emergency room. Initial chest X-ray showed a ruptured left lung cyst (Figure 2).

**Figure 3.**
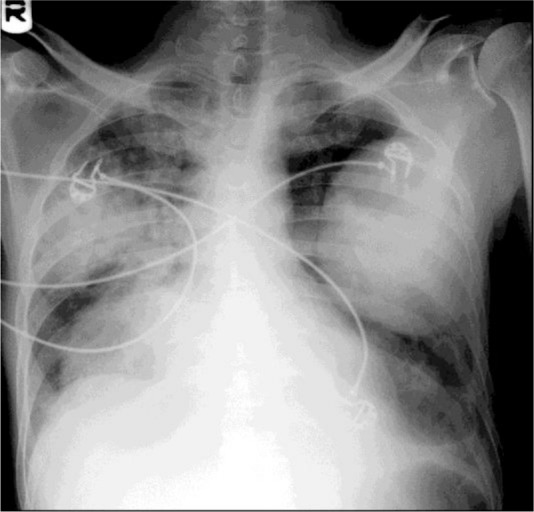
Chest X-ray shows severe pneumonitis with corresponding cysts.

**Figure 4.**
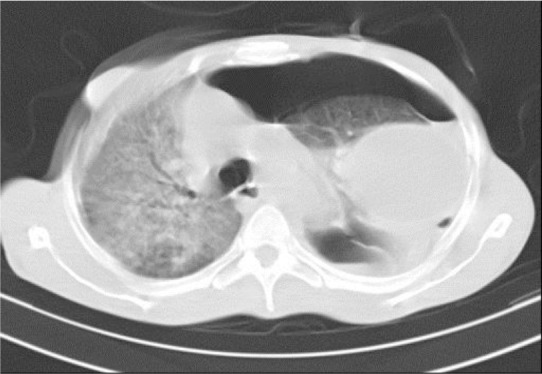
Tension pneumothorax in CT scan in association with severe pneumonitis.

Antibiotics (ciprofloxacin, vancomycin, and meropenem) were administered intravenously in conjunction with dexamethasone and albendazole that was begun orally. In spite of these treatments respiratory distress worsened and due to severe dyspnea and diagnosis of respiratory insufficiency and empyema the patient was admitted to ICU. At this time chest x-ray revealed severe infiltration and pneumonitis in right lung that was probably due to flooding of the left cyst content after its rupture in to right lung (chemical pneumonitis) (Figure 3). After transferring the patient to ICU, supportive respiratory care for facilitating airway drainage in conjunction with wide spectrum antibiotic was selected for the patient. Due to left-sided pneumothorax (Figure 4) a chest tube was inserted and copious amount of malodorous pus drained. Following that, there was a little progression in respiratory signs and symptoms with continuing degree of dyspnea and productive cough. Again, after four days with worsening of respiratory symptoms and respiratory failure the patient was intubated and placed on mechanical ventilation (sixteenth day after admission), however due to the progression of the infectious process and severe sepsis, the patient died.

## DISCUSSION

Hydatid cyst is an endemic disease in Iran and other Middle East countries. The liver is the most common location of the cyst and the lung is the second most common organ that is involved in this disease (1). Most authors believe that the best treatment for pulmonary hydatid cyst is the surgical evacuation of the cyst, closure of bronchial openings with or without capitonnage (2).

Benzimidazole anthelmintic drugs such as albendazole have been used as a prophylactic drug before surgery for reducing recurrence rate or even as a definitive medical treatment in patients that are not a good candidate for surgery due to widespread involvement or intolerance to surgery (3). Therapeutic responses of pulmonary hydatid cyst to albendazole therapy vary and are unpredictable, even in a particular patient with multiple cysts; as cysts may show different therapeutic responses (4,5). In few patients, the cyst will regress and disappear after treatment but on the other hand, some of them will continue their growth. In addition, there are some reports on increasing incidence of rupturing of pulmonary hydatid cysts following albendazole therapy and consequent complications due to this rupture (6,7).

In our patient, one of the pulmonary cysts ruptured following treatment with albendazole that resulted in pulmonary insufficiency and death. Probably the main explanation for this event was chemical pneumonitis that was induced by the highly allergic content of cyst which affected both lungs, although on chest x-ray right lung was affected more intensely. As mentioned before the therapeutic response of pulmonary hydatid cyst is unpredictable and in this patient, only one of three cysts ruptured following albendazole treatment but this event terminated with a grave outcome for the patient. Anaphylactic shock and even sudden death have been reported after rupturing of pulmonary hydatid cyst (8), although this clinical presentation is an uncommon scenario and most of these patients will recover from this complication without any serious morbidity and mortality (9). In our patient, the ruptured cyst was a giant one and this probably led to vigorous complication and patient death.

Usluer et al. in their experimental study showed that tensile strength of cuticular membrane of pulmonary hydatid cysts would be decreased following administration of albendazole (10) and we also believe that the main cause of this spontaneous rupture of cyst is treatment with albendazole. This idea will highlight the question that (Is it an acceptable strategy to use albendazole as a prophylactic measure in patients with pulmonary hydatid cyst?). Some authors believe that albendazole is prohibited in giant cysts or at least must be limited to a short duration for about 4–7 days before surgery similar to antibiotic prophylaxis before any other surgeries. On the other hand, short duration of albendazole treatment has little effect on the population of scolex and this therapeutic effect will appear after at least ten days following administration of this drug (11,12).

According to increased incidence of pulmonary hydatid cyst rupture following albendazole therapy and not fully recognized effect of the drug in reducing recurrence rate, it is probable that the best-justified policy in the treatment of pulmonary hydatid cyst is immediate surgical evacuation without any further albendazole prophylaxis.

## CONCLUSION

Albendazole has been used as a prophylactic treatment before surgery in patients with pulmonary hydatid cyst for reducing recurrence rate. This drug can cause spontaneous rupture of cysts with some unpredictable complications. Although rupture of cyst can occur in the absence of albendazole treatment we believe that this drug is a potential cause and more investigations are needed to prove it clinically. Therefore, we believe early surgical treatment even without albendazole prophylaxis provides an acceptable strategy for treating these patients.
